# Poster Session II - A229 CHILDHOOD EXPOSURE TO A SIBLING WITH CROHN’S DISEASE ALTERS GUT MICROBIOME AND INCREASES DISEASE SUSCEPTIBILITY

**DOI:** 10.1093/jcag/gwaf042.229

**Published:** 2026-02-13

**Authors:** R Chen, H Kim, M Bushra, Q Li, D Turner, H Steinhart, H Q Huynh, K Jacobson, A Griffiths, W Turpin, K Croitoru, S Lee

**Affiliations:** IBD Center and Zane Cohen Centre for Digestive Diseases, Lunenfeld-Tanenbaum Research Institute, Toronto, ON, Canada; Korea University, Seongbuk-gu, Seoul, Korea (the Republic of); Mount Sinai Hospital, Toronto, ON, Canada; IBD Center and Zane Cohen Centre for Digestive Diseases, Lunenfeld-Tanenbaum Research Institute, Toronto, ON, Canada; The Hebrew University of Jerusalem, Jerusalem, Jerusalem District, Israel; Mount Sinai Hospital, Toronto, ON, Canada; Stollery Children’s hospital, University of Alberta, Edmonton, AB, Canada; BC Children’s Hospital, Vancouver, BC, Canada; The Hospital for Sick Children, Toronto, ON, Canada; IBD Center and Zane Cohen Centre for Digestive Diseases, Lunenfeld-Tanenbaum Research Institute, Toronto, ON, Canada; IBD Center and Zane Cohen Centre for Digestive Diseases, Lunenfeld-Tanenbaum Research Institute, Toronto, ON, Canada; IBD Center and Zane Cohen Centre for Digestive Diseases, Lunenfeld-Tanenbaum Research Institute, Toronto, ON, Canada

## Abstract

**Background:**

Previous studies have reported that sibling pairs share a similar gut microbiome, especially during childhood.

**Aims:**

We hypothesized that childhood exposure (as opposed to adult exposure) to a sibling with Crohn’s disease (CD) may impact the gut microbiome and ultimately, increase disease susceptibility.

**Methods:**

In the GEM Project cohort, which prospectively followed 3,126 healthy siblings of CD patients, we assessed the association between “childhood exposure” to CD (defined as having a sibling diagnosed with CD before the age of 18) and future onset of CD. The association between childhood (vs. adult) exposure and risk of CD was validated in an independent nationwide dataset from South Korea (n = 50 million). Cross-sectional logistic regression in the GEM cohort assessed the association between childhood exposure to CD and baseline biomarkers, including fecal calprotectin (FCP), and 16s rRNA-based gut microbiome composition. An integrative risk model combining childhood exposure, FCP, and specific microbial communities was trained (Canadian sub-cohort) and validated (sub-cohort from outside Canada) in the GEM project.

**Results:**

In the GEM cohort, 2,265 siblings (72.5%) had childhood exposure to siblings with CD. Childhood exposure, compared to adult exposure, was associated with four-fold higher risk of developing CD (adjusted HR (aHR) [95% CI]: 4.00 [1.83-8.75]; P = 5.3 × 10^-4^), after adjusting for sex, country, ethnicity, parental history of CD and CD-polygenic risk score. Similarly, in the South Korean dataset, childhood exposure was an independent risk factor of developing CD, with an aHR (95%CI) of 2.54 (1.70-3.81; P = 6.0 × 10^-6^). 33 microbial genera were differentially abundant in childhood-exposed group (vs adult exposure) in the GEM. Mediation analysis revealed that *Lachnospira, Roseburia* and *Colidextribacter* partially mediates the effect of childhood exposure on CD onset. The integrated model identified a high-risk subset characterized by childhood exposure, microbial community (Endotype) of increased *Blautia* or *Prevotella*, and FCP>100 ug/g, with a 10-year cumulative incidence of CD of 22.5% (training) and 19.8% (validation cohort).

**Conclusions:**

Childhood exposure to a sibling with CD is associated with 4-fold increased risk of developing CD. Gut microbial community profiles not only help refine risk stratification among these siblings but also suggest a potential role of the gut microbiome as a mechanistic link between early-life exposure to a sibling with CD and increased disease susceptibility.

**Funding Agencies:**

CCC, CIHRHemsley Charitable Trust

